# Physiological relevance of post-translational regulation of the spindle assembly checkpoint protein BubR1

**DOI:** 10.1186/s13578-021-00589-2

**Published:** 2021-04-23

**Authors:** Celia R. Bloom, Brian J. North

**Affiliations:** Biomedical Sciences Department, Creighton University School of Medicine, 2500 California Plaza, Omaha, NE 68178 USA

**Keywords:** Post-translational modifications, BubR1, Mitosis, Acetylation, Phosphorylation, Ubiquitination, SUMOylation

## Abstract

BubR1 is an essential component of the spindle assembly checkpoint (SAC) during mitosis where it functions to prevent anaphase onset to ensure proper chromosome alignment and kinetochore-microtubule attachment. Loss or mutation of BubR1 results in aneuploidy that precedes various potential pathologies, including cancer and mosaic variegated aneuploidy (MVA). BubR1 is also progressively downregulated with age and has been shown to be directly involved in the aging process through suppression of cellular senescence. Post-translational modifications, including but not limited to phosphorylation, acetylation, and ubiquitination, play a critical role in the temporal and spatial regulation of BubR1 function. In this review, we discuss the currently characterized post-translational modifications to BubR1, the enzymes involved, and the biological consequences to BubR1 functionality and implications in diseases associated with BubR1. Understanding the molecular mechanisms promoting these modifications and their roles in regulating BubR1 is important for our current understanding and future studies of BubR1 in maintaining genomic integrity as well as in aging and cancer.

## Background

Aging is one of the greatest risk factors for the development of a variety of diseases, including cancer [1]. While it has been long known that aging is a key contributor to disease, exploration of the molecular basis of the interrelationship between aging and disease has made notable progress over the past two decades. BubR1, which is a regulator of genomic integrity, may lie at the interface of aging and cancer. BubR1 is a putative serine-threonine protein kinase with a variety of critical cellular functions, most of which are largely mitotic [2, 3]. It is a component of the spindle assembly checkpoint (SAC) where its function is strictly regulated to ensure proper chromosome segregation through its involvement in coordinating kinetochore-microtubule interactions, chromosome migration and alignment, and anaphase inhibition [4–6]. Loss or mutation of BubR1 is associated with aneuploidy, which is often accompanied by pathological consequences such as cancer, premature/accelerated aging, and mosaic variegated aneuploidy (MVA) [7–11]. Furthermore, BubR1 levels progressively diminish with age. This decline has been shown to be directly involved in the aging process through control of cellular senescence [12–14]. Given both this and its involvement in maintaining genomic integrity, BubR1 may be a crucial link between aging and increased cancer risk.

The most well-described functions of BubR1 take place during chromosome migration and alignment in prometaphase [15]. BubR1 serves both to facilitate kinetochore-microtubule interactions that comprise the dynamic process of error correction and chromosome migration, and to prevent anaphase from commencing until all chromosomes are aligned at the metaphase plate and attached properly to bipolar spindles. Following chromosome condensation and nuclear envelope breakdown, the presence of exposed kinetochores serves as a ‘wait anaphase’ signal that triggers the SAC [16]. BubR1 is recruited to kinetochores as early as prophase to prevent anaphase from occurring prematurely, thus allowing time for microtubules to find and properly attach kinetochores to guide chromosomes to the metaphase plate. Here, they achieve precise alignment and biorientation, which signals that the cell is ready for anaphase, and the SAC response can be silenced.

Mitosis is a highly regulated process consisting of a myriad of cooperative mechanisms, many of which require dynamic control of key mitotic regulators to keep the cell in such a state that allows it to respond rapidly to new circumstances. For example, when all chromosomes are properly aligned and attached, the SAC must be quickly silenced to allow for complete initiation of anaphase [17]. Repeated opposing activities of key mitotic players contribute to the ability of the cell to accomplish this. BubR1 is a central component of these mechanisms where its mitotic functions are often dichotomized, as it plays roles in both anaphase inhibition and in kinetochore-microtubule interaction dynamics during error correction and chromosome migration. These roles are distinguishable from each other, and specific mutations may affect one of these functions of BubR1 while leaving the other fully intact. BubR1 levels increase dramatically just before mitosis, and following its recruitment to kinetochores, one pool becomes designated as having a kinetochore-microtubule function, while another pool contributes to anaphase inhibition by forming the mitotic checkpoint complex (MCC) with Cdc20, Bub3 and Mad2 [18, 19].

Error correction and SAC responses are dictated by two properties of kinetochore-microtubule interactions: (1) the attachment state of kinetochores to microtubules, and (2) tension across the kinetochores between sister chromatids [20–22]. Unattached kinetochores and lack of tension (which by nature are tightly linked) are both able to trigger BubR1 activation and the SAC response, and their resolution requires the activity of error correction machinery at kinetochores. These properties are contingent on the activity of numerous mitotic proteins and other factors, such as the kinetic phenomena of microtubule pushing and pulling forces [23]. Importantly, much of BubR1 post-translational control is dictated by these processes. For instance, a post-translational modification (PTM) may be sensitive to attachment state but not tension, or vice versa. Related to this, throughout prometaphase, kinetochores and microtubules are repeatedly attached and detached [24], a mechanism that contributes to chromosome oscillations and migration [25–27] and that heavily relies on counterbalancing PTMs mediated largely by mitotic kinases and phosphatases [28]. These concepts will be discussed in greater detail in the context of each phosphorylation or other PTM.

Like many proteins, BubR1 activity and abundance are controlled by PTMs such as phosphorylation, acetylation, and ubiquitination. PTMs are a crucial regulatory mechanism in fine-tuning BubR1 activity throughout the cell cycle, especially during mitosis [29]. Here, we discuss PTMs of BubR1 and their critical role in regulating BubR1 functionality to maintain genomic integrity. Furthermore, we will discuss these modifications and their implications in diseases associated with BubR1. Understanding the molecular mechanisms promoting these modifications and their roles in BubR1 function is important for our current understanding and future studies of BubR1.

## Domain organization of BubR1

**Fig. 1:**
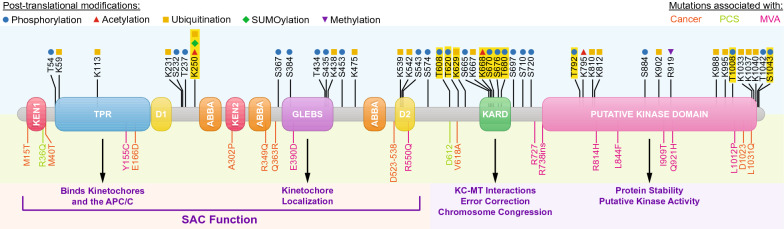
Domain organization of BubR1. The main functions associated with the various domains are highlighted. Domains regulating BubR1 degradation include KEN-Boxes (KEN1 and KEN2), Destruction Boxes (D1 and D2), and ABBA (named for found in Cyclin A, BubR1, Bub1, and AMC1) domains. Domains responsible for protein–protein interactions include tetratricopeptide repeat motif (TPR), Gle2-binding-sequence (GLEBS), and kinetochore attachment regulatory domain (KARD). The C-terminal region of BubR1 contains the putative kinase domain. Shown are characterized and experimentally observed PTMs (above) and location of mutations found in BubR1 relating to cancer, PCS, and MVA (below). PTMs highlighted in yellow have been experimentally studied and are discussed in this review

The organization of the functional domains of BubR1 support its biological functions during mitosis. The overall domain structure of BubR1 is segregated spatially across the protein and highlights BubR1s dual role during mitosis (Fig. 1). It has been frequently observed that the N-terminal region of BubR1 contributes primarily to its SAC function [30], while the C-terminal region is responsible for its kinetochore-microtubule function. The identities and structure–function relationships associated with the various domains of BubR1 are still being elucidated (Table 1).

**Table 1:** Domain structure function relationship

Domain	Function
KEN1	APC/C degron; Binds Cdc20 MCC assembly: Brings together BubR1, Bub3, Mad2, Cdc20
TPR motifs	Binds KNL1 protein (MELT motifs) on kinetochores Anaphase inhibition: Binds an E2 binding domain on APC/C
D box 1	APC/C degron; Binds Cdc20 at its D box recognition domain, preventing recognition of D box-containing substrates
ABBA motif (A1)	Binds Cdc20
KEN2	APC/C degron; Binds Cdc20 APC/C inhibition: Blocks substrate recruitment to APC/C
ABBA motif (A2)	Binds Cdc20
GLEBS; Bub3-binding	BubR1 recruitment to kinetochores via Bub3 binding
ABBA motif (A3) (Phe box; IC20BD)	Binds Cdc20-APC/C Unknown the extent of its importance
D box 2	APC/C degron; Binds Cdc20 at its D box recognition domain, preventing recognition of D box-containing substrates
KARD	PP2A-B56 recruitment to kinetochores
Kinase domain	Potentially catalytic: autophosphorylation, targeted phosphorylation of other mitotic proteins May be involved in BubR1 protein stability

The N-terminal region of BubR1 contains many APC/C degron domains, including multiple KEN boxes, destruction boxes (D boxes), and ABBA motifs (also known as Phe boxes) (Fig. 1). These domains allow BubR1 to interact with the APC/C through its Cdc20 recognition subunit to physically block APC/C activation [31, 32]. The N-terminus also harbors the GLEBS domain, which serves as a binding region for Bub3 and is important for BubR1 recruitment to kinetochores [30, 33, 34], and a series of TPR motifs that play roles in kinetochore binding as well as interacting with the APC/C (Fig. 1) [35, 36]. Meanwhile, the C-terminal half of BubR1 is primarily responsible for its kinetochore-microtubule function (Fig. 1). The kinetochore attachment regulatory domain (KARD) is found within the C-terminal half and is critical to the kinetochore-microtubule monitoring activity of BubR1 [37]. The KARD is highly phosphorylated during mitosis while the SAC is active, and subsequent dephosphorylation of this domain is a key step in checkpoint silencing. In addition, extending the remaining length of the C-terminus is a putative kinase domain. Whether this domain is indeed catalytic has remained unclear. Various lines of evidence suggest that it remains non-functional, while recent studies support a role for the BubR1 kinase domain in regulating its stability and abundance as well as catalytically acting upon multiple targets during mitosis [38]. Importantly, post-translational modifications in and around the kinase domain of BubR1 have demonstrated significance in regulating BubR1 function and abundance. Furthermore, mutations of BubR1 are associated with MVA and are often clustered in the kinase domain, suggesting a potential role for this region in mediating disease pathology (Fig. 1).

Many of BubR1s functional domains in contain key post-translationally modified amino acids (Fig. 1). The occurrence of PTMs residing within, or in close proximity to, key functional domains of BubR1 strongly implicates post-translational regulation as a key mechanism controlling BubR1 function.

## Regulation of BubR1 by PTMs

Post-translational modifications refer to changes that take place on a protein following its synthesis. A protein may not be fully mature until it has been post-translationally modified, or its function may be linked directly to the modification. There are over 200 known modifications that can occur on proteins, which may involve the addition of chemical groups such as a phosphoryl or acetyl; lipids such as myristoyl or palmitoyl; sugars such as glycosyl; or polypeptides such as ubiquitin or SUMO on the termini or amino acid side chains of the target protein [39, 40]. As a result, subjecting proteins to various PTMs can yield drastically different consequences, including but not limited to regulating activity, binding/complex formation, and stability.

**Fig. 2:**
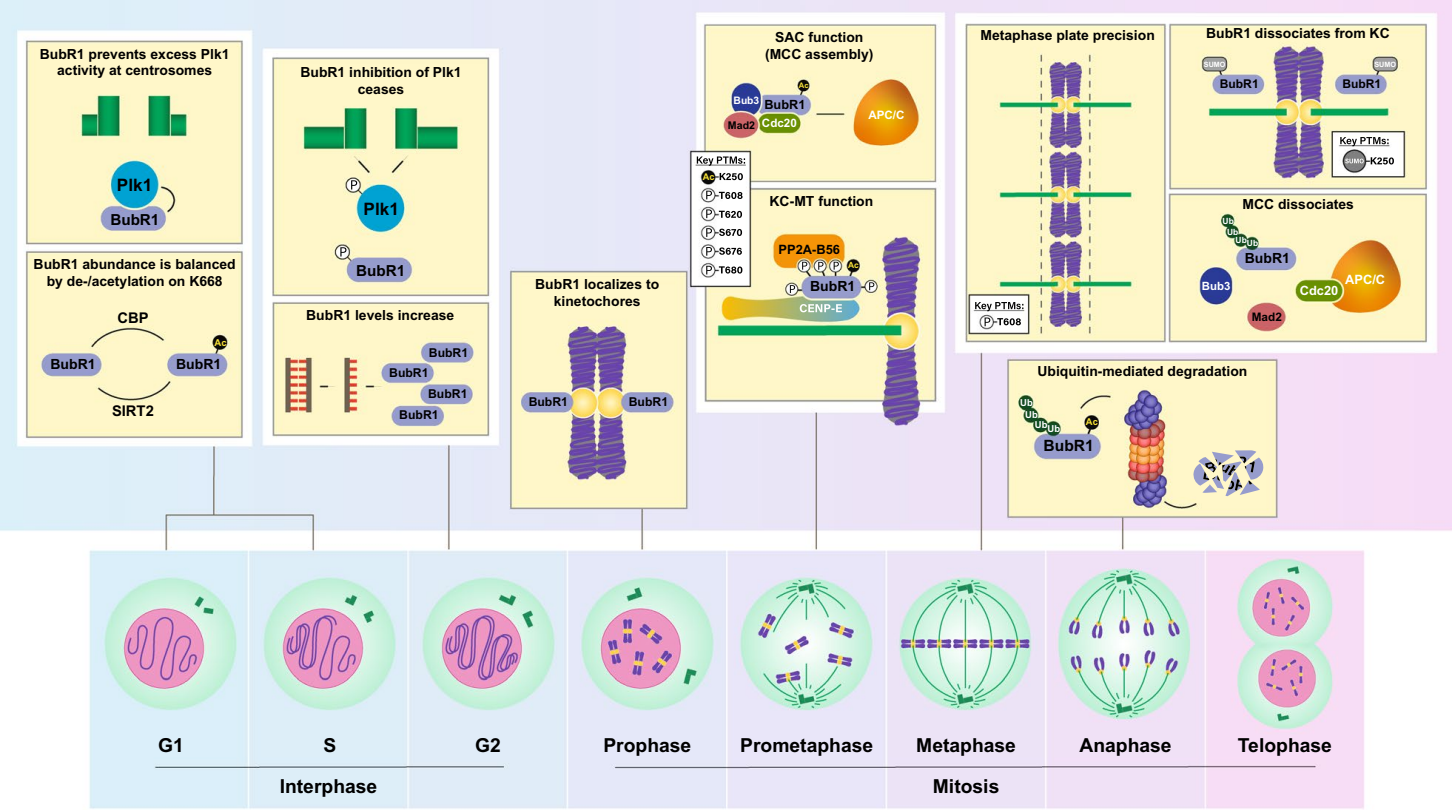
Cell cycle of BubR1 functionality and abundance. Key regulatory PTMs that occur on BubR1 during interphase, as well as the various stages of mitosis, are shown

Numerous studies have identified PTMs on BubR1 such as phosphorylation, acetylation, ubiquitination, and SUMOylation, each of which has been demonstrated to control BubR1 function in a cell cycle-dependent manner (Figs. 1 and 2). As a predominantly mitotic protein with timing-specific functions, BubR1 must be strictly regulated temporally and spatially to modulate MCC and kinetochore-microtubule turnover. Notably, the MCC is constantly disassembled and reassembled during an active SAC response. This reversibility is important in allowing the rapid silencing of the SAC upon successful chromosome alignment and biorientation. In addition, key kinases and phosphatases involved in kinetochore-microtubule interactions are constantly opposing the activity of each other throughout chromosome migration and the SAC response. These “tug-of-war” phenomena require swift activation and silencing of protein activity, which is efficiently mediated through reversible PTMs.

## Phosphorylation of BubR1

One of the most common PTMs, phosphorylation is a reversible process that takes place primarily on serine, threonine, and tyrosine residues. Addition of the phosphoryl group is accomplished by enzymes called kinases, while removal of the group is mediated by phosphatases. During interphase, BubR1 predominantly exists in a dephosphorylated state [38, 41], but during mitosis, it is phosphorylated on numerous sites, promoting its activity especially in the context of kinetochore-microtubule interactions to contribute to error correction and chromosome congression. Most of the characterized BubR1 phosphorylation events are directly accomplished by the mitotic kinases Plk1 and Cdk1. While the kinase Mps1 may phosphorylate BubR1 directly, it is more likely to regulate BubR1 PTMs indirectly through controlling upstream modulators [42, 43]. Unlike the kinases involved in BubR1 modification, very little is known about the phosphatases that remove the phosphoryl groups, although PP1 and PP2A-B56 have been implicated [44–46].

Different aspects of BubR1 activity are known to be regulated via phosphorylation events on multiple residues as described below.

### T620

Cell cycle regulation is orchestrated by the temporal fluctuations of cyclins and their control of cyclin-dependent kinases (CDKs) [47]. The start of mitosis is marked by an increase in abundance of Cyclin B1, which binds to and activates Cdk1. Cyclin B1-Cdk1 phosphorylates numerous substrates involved in mitotic progression [48, 49], dramatically reorganizing the cellular makeup and committing the cell to progress through mitosis. Among the Cyclin B1-Cdk1-mediated PTMs on BubR1 is phosphorylation at T620, which initiates a cascade of events that dictate BubR1s function in regulating kinetochore-microtubule stability [28]. This PTM creates a docking site for Polo-like kinase 1 (Plk1), a multifunctional kinase with roles in many cellular processes and events, including mitosis. Plk1 bound to BubR1 then phosphorylates Ser/Thr residues within the KARD, particularly S676 and T680 [28, 37], which is critical for promoting kinetochore attachment to microtubules by recruiting the phosphatase PP2A-B56. Collectively, these events support the kinetochore-microtubule function of BubR1 in chromosome congression and error correction.

Docking of Plk1 on BubR1 involves the interaction between the Polo-Box domain (PBD) on Plk1 and a motif on BubR1 containing phosphorylated T620. PBDs are specialized domains that recognize specific motifs containing phosphoresidues, which is characterized by a Ser-pThr-Pro (STP) motif [50]. The T620 residue lies within such a motif on BubR1 that is also found in the related Bub1 kinase [51], which interacts with Plk1 in a similar manner. Therefore, phosphorylated T620 likely serves as a priming site for Plk1 docking [28]. Cdk1 and Plk1 kinases are both capable of phosphorylating residues within STP motifs to create PBD docking sites [28].

Phosphorylation at T620 is important for the kinetochore-microtubule stabilization activity of BubR1 but does not appear to influence its SAC function [28]. Cells with BubR1 amino acid substitutions that prevent T620 phosphorylation display defects in kinetochore-microtubule attachments and chromosome migration. Since T620 phosphorylation is not required for BubR1 to inhibit anaphase, such cells retain the ability to maintain mitotic arrest as long as the kinetochore-microtubule errors remain unresolved [28]. The loss of the ability to resolve errors in T620 phosphorylation-deficient mutants thus leads to prolonged mitosis that eventually ends in abnormal mitotic exit, such as mitotic slippage, leading to aneuploidy [28]. Thus, its role in kinetochore-microtubule stability through the initiation of a mitotic phosphorylation cascade makes T620 phosphorylation a critically important BubR1 PTM during mitosis.

### S670, S676 and T680 within the kinetochore attachment regulatory domain (KARD)

The KARD, spanning residues 665 to 682, is a highly conserved domain that is critical for proper BubR1 functioning [37]. PTMs occurring within the KARD promote BubR1-mediated kinetochore-microtubule stabilization activity. During mitosis, the KARD is phosphorylated at residues S670, S676, and T680, whose modified states determine BubR1 activity and mitotic progression. Loss of this region results in severe chromosome misalignment and missegregation, leading to aneuploid daughter cells [37].

KARD phosphorylation is vital in the kinetochore-microtubule interaction dynamics that comprise error correction and chromosome migration to the metaphase plate. As microtubules extend from spindle poles to make contact with kinetochores, BubR1 is phosphorylated first at T620, then at KARD residues S670, S676, and T680 [37, 52, 53]. These PTMs within the KARD create a docking site for the PP2A-B56 phosphatase, which binds to BubR1 and promotes kinetochore attachment to microtubules by dephosphorylating kinetochore proteins [52, 54, 55]. These kinetochores, although now attached, may still be misaligned and therefore lack tension. Aurora B kinase responds to a lack of kinetochore tension by phosphorylating key proteins on the kinetochore, counteracting the activity of PP2A-B56, and disrupting kinetochore-microtubule interaction [52, 55]. Repetition of these opposing activities yields the dynamic of chromosome migration [27, 56] and continues until all chromosomes are correctly aligned at the metaphase plate, bipolarly attached to microtubule fibers, and generating sufficient tension to silence the error correction machinery at kinetochores. Importantly, Aurora B silencing may occur by both tension-dependent and tension-independent mechanisms [57]. Any attachment errors that are generated during this process, such as syntelic (two kinetochores of a given chromosome are attached to microtubules from the same spindle pole) or monotelic (one of the two sister kinetochores of a chromatid pair is attached to one spindle pole while the other sister kinetochore remains unattached), are also corrected by this KARD-dependent mechanism. Phosphorylation of the BubR1 KARD is essential for PP2A-B56 recruitment and therefore is a critical component in kinetochore-microtubule interactions, chromosome migration, and alignment. The KARD is a major site of rapid and reversible modification, contributing to the fine-tuning of this function of BubR1. However, the dynamics of BubR1 modification in and around the KARD and its significance in controlling genomic stability and suppressing tumorigenesis are complex and continue to be studied.

The phosphorylated residues within the KARD are often discussed collectively as Plk1 targets, though S670 phosphorylation appears to be Plk1-independent and may instead be targeted by Cdk1 [42]. Importantly, multiple kinases acting on these residues may reflect the differential sensitivities of these PTMs. For instance, S670 phosphorylation is dependent on kinetochore-microtubule attachment state [43], while S676 and T680 phosphorylation are sensitive to kinetochore tension [37, 45]. Plk1 phosphorylation of the KARD (and potentially other residues) has been demonstrated to be localization dependent [28]. BubR1, and therefore Plk1, must both localize to the kinetochores in order for BubR1 mitotic hyperphosphorylation to occur.

**Fig. 3:**
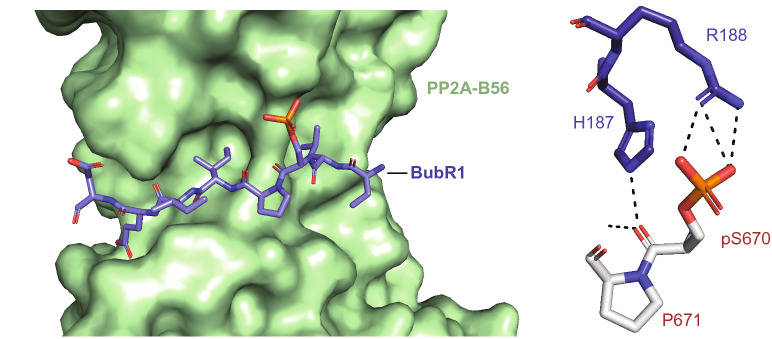
BubR1 KARD domain binding to PP2A-B56. Structure of BubR1 KARD peptide phosphorylated at S670 bound to PP2A-B56. Phosphorylated S670 forms multiple contacts with R188 of PP2A-B56

BubR1 binds to the phosphatase PP2A-B56 specifically through the B56 subunit [45, 52]. The KARD encompasses the B56 binding site, identified as a LxxIxE motif contained within residues 668–675 (K**L**SP**I**I**E**D) (Fig. 3) [45]. Among the residues in this motif, four sites in particular, K668, L669, I672 and E674, were identified to physically interact with PP2A-B56, contributing to its binding to BubR1 [45, 52]. While phosphorylation of the KARD is known to influence PP2A-B56 recruitment and/or binding, the individual roles of each PTM in this context remain unclear. Multiple studies have investigated the contributions of the phosphorylation states of S670, S676, and T680 to BubR1-B56 binding, given their location relative to the B56 binding motif. The importance of p-S670 in BubR1-B56 binding has been demonstrated recently [52]. Additional studies have shown both p-S670 and p-S676 as being important for B56 binding to the KARD, while a role for T680 remains unclear [37, 45]. Therefore, further studies elucidating the relative contributions and responsiveness of each of these phosphorylation sites within the KARD are necessary to fully appreciate the role of this phosphodomain in BubR1s ability to control dynamic error correction activity and establish proper metaphase chromosome alignment.

#### S670

Unlike Plk1 targets S676 and T680, S670 is phosphorylated by Cdk1 [42]. It is postulated that Mps1 may also phosphorylate S670, however there is a lack of evidence of a direct interaction, thus it may influence BubR1 KARD phosphorylation indirectly [42]. Phosphorylation at S670 occurs specifically in response to unattached kinetochores, as p-S670 levels increase at unattached kinetochores and decrease in the presence of attached kinetochores even if there remains a lack of tension [43]. Thus, S670 phosphorylation state is dependent upon kinetochore-microtubule attachment state at any given moment, highlighting the critical importance of a rapid, reversible PTM mechanism throughout the process of chromosome congression. S670, along with S543 and S1043, was shown to be gradually dephosphorylated during the process of mitotic exit, although the phospho-signals that persisted into anaphase may be a reflection of cytoplasmic BubR1 as opposed to kinetochore-bound BubR1 [43]. Unphosphorylatable mutants of S670 demonstrated a greater incidence of lagging chromosomes upon anaphase onset as compared to wild type and S670 phosphomimetic mutants, suggesting that loss of p-S670 results in unresolved kinetochore-microtubule attachment deficits. Importantly, cells expressing S670 unphosphorylatable mutants retained the ability to arrest in mitosis, indicating that this PTM is more important in kinetochore-microtubule stability and is not directly required for BubR1 SAC function [43]. Phosphorylation of S670 due to lack of attachment may be explained by the activity of Cyclin B1-Cdk1. As one of the key early mitotic regulatory kinases, Cyclin B1-Cdk1 may be responding to the earliest initial unattached kinetochores of newly condensed chromosomes immediately following nuclear envelope breakdown and entry into prophase. Therefore, whether T620 or S670 is modified first upon mitotic entry remains unclear.

Recent structural data reveal a potentially important role for S670 in regulating the interaction between the BubR1 KARD domain and PP2A-B56. Crystal structure of PP2A-B56 in complex with a phosphorylated S670 KARD peptide reveals multiple electrostatic and H-bond interactions between p-S670 in BubR1 and R188 of PP2A-B56 [58], further emphasizing the role of this site in regulating the interaction between BubR1 and PP2A-B56 (Fig. 3).

#### S676

In contrast to S670, S676 is modified by docked Plk1 in response to a lack of kinetochore tension [45]. Unattached kinetochores do not generate tension, thus tightly linking these two properties of kinetochore-microtubule interactions. However, attached kinetochores can still lack tension if they are misaligned or erroneous in some way. For example, when both kinetochores attach to microtubules extending from the same spindle pole (i.e., syntelic attachments), tension-dependent scenarios such as these promote S676 [43] and T680 phosphorylation [37, 45], but may not promote S670 phosphorylation. The ability of BubR1 to be tension-sensitive is crucial not only during the process of chromosome congression where correct bipolar attachments do not yet generate tension due to being misaligned but also in the correction of certain tension-lacking attachment errors. In other words, this tension-responsive mechanism reinforces the ability of BubR1 to correct errors in the event that all kinetochores are attached to microtubules but may not be oriented correctly. S676 is phosphorylated during prometaphase and dephosphorylated at metaphase upon generation of tension across sister kinetochores [28]. Furthermore, phosphorylation at S676 has been shown to require prior phosphorylation at T620, indicating the importance of Plk1 binding to p-T620 to promote KARD phosphorylation [28].

#### T680

Also modified by Plk1, T680 is a critical component of the KARD and may function in a similar manner as S676 as tension-sensitive and unaffected by kinetochore attachment state [37]. Phosphorylation of T680 has been shown to occur following nuclear envelope breakdown [37]. Furthermore, phospho-deficient mutation of T680 abolished BubR1 activity to a greater extent than both S670 and S676, suggesting that this residue may be the most essential of the three sites [37]. However, this is contradictory to data regarding PP2A-B56 binding to BubR1, which suggested that T680 may not be required for the direct binding between B56 and BubR1 [45]. However, given the demonstrated importance of p-T680 to BubR1 function, there may be an additional contribution of p-T680 in KARD-mediated error correction that is independent of regulating PP2A-B56-BubR1 binding, which requires further elucidation [45].

Phospho-deficient mutations of each of these KARD residues alone results in a relatively minor defect in alignment capacity, with mutation of T680 being the most significant of the three [37]. However, mutations at all three of these residues together abolish chromosome alignment in a manner that phenocopies the deletion of BubR1. Closer examination shows that such mutants, in the same manner as a ΔKARD mutant, are unable to form stable kinetochore-microtubule attachments. This is likely a direct result of the inability of BubR1 to recruit PP2A-B56 to kinetochores [37]. Therefore, the BubR1-PP2A-B56 interaction that results from dynamic phosphorylation of the BubR1 KARD promotes and stabilizes kinetochore-microtubule interactions by balancing Aurora B activity at kinetochores throughout the process of chromosome migration and alignment [37]. However, there is still much to learn regarding the differential sensitivities of each of these residues to unravel their full significance in regulating BubR1 function.

### T608

Phosphorylation of BubR1 at T608 has been identified as an important PTM, and its discovery and study have contributed to the enduring debate over whether BubR1 has a functional kinase domain. It has been suggested that p-T608 may be a result of BubR1 autophosphorylation, but there are also theories that T608 is targeted by Plk1 [37]. Multiple potential roles for p-T608 have been identified, including metaphase plate precision and the resolution of polar chromosomes [59]. These functions are accomplished through the mechanistic influence of BubR1 on Aurora B activity as well as Mad1–Mad2 recruitment to kinetochores [59]. Immunofluorescence studies demonstrated phosphorylated T608 is enriched at kinetochores during prometaphase but diminished at metaphase after chromosomes were fully aligned. Unphosphorylatable mutants of T608 produce defects in chromosome alignment and a weakened SAC [59].

Phosphorylation of BubR1 at T608 appears to also be dependent on CENP-E, a motor protein with important roles in chromosome migration and alignment. In particular, CENP-E has been shown to be crucial in the migration of polar chromosomes (those that are located in close proximity of either spindle pole rather than near the metaphase plate following nuclear envelope breakdown) and also contributes to the precision of metaphase plate alignment [59, 60]. Cells depleted of CENP-E display a higher incidence of nonmigrating, mono-oriented polar chromosomes, and also reduced ability of non-polar chromosomes to form a distinct metaphase plate [60]. Interestingly, T608 phosphorylation on BubR1 appears to be related to certain CENP-E functions. Recent results confirmed that CENP-E and BubR1 interact through a C-terminal helix within the BubR1 kinase domain which may be necessary for CENP-E recruitment to the kinetochore via BubR1 [61]. It has also been postulated that the interaction between BubR1 and CENP-E through the BubR1 kinase domain stimulates BubR1s supposed kinase activity, allowing it to phosphorylate, among other substrates, itself at T608 [59]. Cells with kinase-dead BubR1 or with unphosphorylatable T608 phenocopy cells with CENP-E depletion [59, 60, 62], suggesting a tight link between CENP-E function and the phosphorylation state of T608 on BubR1, as well as implicating the BubR1 kinase domain as having a role in these established mitotic processes, whether it is functionally catalytic or not.

Although mitosis is well-established to be an all-or-nothing event, the SAC has been described as a graded response [63, 64], wherein the amount of functional MCC available to inhibit the APC/C is sensitive to the presence of unattached/tensionless kinetochores. This derives somewhat of a paradox, as a cell with a single error can be in greater danger of chromosome missegregation than one with many errors, depending on the circumstances, because there is less APC/C inhibition machinery present. In normal cells, a single unattached kinetochore is sufficient to initiate and maintain a SAC response. CENP-E is required for SAC function in instances of one or a few unattached kinetochores (often polar chromosomes) [11], a process that also appears to require phosphorylation of BubR1 on T608 [59]. Cells absent in p-T608 have roughly the same duration of mitosis as wild type cells, implying that this PTM is not essential for mitotic timing under normal conditions. However, such cells often enter anaphase with one or a few unresolved polar chromosomes [59], suggesting that these remaining errors are not sufficient to initiate or maintain a robust SAC response without p-T608. Consistent with this notion, loss of T608 phosphorylation resulted in reduced levels of Mad2 at kinetochores, indicating a weakened SAC response [59]. Furthermore, the amount of time spent in agent-induced prolonged mitotic arrest was reduced in cells expressing unphosphorylatable T608 compared to cells with wild type BubR1. Interestingly, polar chromosomes accumulate more Mad2 than chromosomes within the spindle, as polar chromosomes are a considerable danger to genomic integrity and the cell must quickly respond to their presence. These results indicate that p-T608 not only contributes to the ability of polar chromosomes to migrate, but also to the ability of a cell to maintain a prolonged SAC response in the presence of one or a few errors which are often attributed to polar chromosomes. This is significant biologically as it serves as an example of a direct link between BubR1s error correction function and its anaphase inhibition function.

BubR1 and CENP-E are thought to colocalize at kinetochores prior to T608 phosphorylation [59, 61, 65]. The presence of kinetochore–microtubule attachment errors stimulates Aurora B activity to phosphorylate KMN proteins and CENP-E, causing destabilization of the kinetochore-microtubule attachments [66, 67]. At unattached kinetochores, the interaction between CENP-E and BubR1 promotes the putative kinase activity of BubR1, allowing it to autophosphorylate T608 [59]. This event signals the recruitment of Mad1-Mad2 complexes, contributing to the SAC response. Through a yet undiscovered mechanism, p-T608 also promotes Aurora B-mediated phosphorylation of the KMN protein Ndc80, contributing to kinetochore–microtubule destabilization [59]. Upon bioriented microtubule capture by CENP-E, the kinase activity of BubR1 ceases, and protein phosphatase 1 (PP1) is recruited to kinetochores, which may be responsible for T608 dephosphorylation. Dephosphorylation of BubR1 leads to a decrease in Aurora B activity, promoting stable kinetochore-microtubule connections, and reduces levels of Mad1–Mad2 at kinetochores, thereby curbing the SAC response. In cells without CENP-E, BubR1 is present at kinetochores with unphosphorylated T608, which reduces Aurora B-mediated Ndc80 phosphorylation and the destabilization of kinetochore-microtubule connections. This results in the inability of the cell to resolve syntelic attachments that frequently occur in polar chromosomes. As a result, cells absent of CENP-E, and therefore p-T608, have persistent polar chromosomes that are unable to migrate [57, 59]. However, this pathway needs to be further explored with respect to other key mitotic proteins, such as PP2A-B56 and Plk1.

While it has not been clearly demonstrated that PP1 dephosphorylates T608, there is additional evidence supporting this notion. PP1 interacts with CENP-E in a manner that opposes Aurora B activity and has been shown to be involved in the “tug-of-war” or “push–pull” dynamic of chromosome congression [68]. This Aurora B/PP1/CENP-E pathway also contributes to the oscillations that occur at metaphase so that chromosomes remain aligned at the metaphase plate until anaphase onset, thus promoting metaphase plate precision [27, 68, 69], a process also involving p-T608 on BubR1 [59]. In addition to its involvement in chromosome congression, PP1 is also a prominent SAC silencer. It has been suggested that CENP-E effectively delivers PP1 to the kinetochore [68], which may lead to the dephosphorylation of BubR1 at T608, thus reducing Aurora B kinase activity to maintain stabilized connections and subsequent removal of Mad1-Mad2 from kinetochores to contribute to SAC silencing. These various points of evidence support the possibility of PP1 dephosphorylating BubR1 at T608 during both chromosome congression and SAC function.

The observation that this self-executed PTM promotes Aurora B activity is noteworthy, as total BubR1 depletion is generally considered to result in increased Aurora B activity. This suggests that p-T608 exists, at least in part, to support Aurora B function. As BubR1 is well-known for its cofunction with PP2A-B56 to antagonize Aurora B activity, this PP1/BubR1 pathway further highlights the importance of PTMs fine-tuning BubR1 spatial and temporal function during mitosis. Multiple PTMs on the same protein having opposing functions is a strong indicator of the presence of a finely controlled biological switch. This concept introduces a different perspective from which to examine the role of multiple pathways converging on BubR1 during mitosis to control kinetochore-microtubule dynamics during chromosome congression.

Although phosphorylation of BubR1 at T608 is an important regulator of BubR1 function, it remains to be clarified whether it is indeed the result of autophosphorylation or through an alternative mechanism. The functionality of the BubR1 kinase domain has been long debated in literature. While it was initially hypothesized to be a pseudokinase [70], recent results suggest the BubR1 kinase domain may be catalytic, supporting the proposition that T608 is a site of autophosphorylation [71]. Of note, CENP-E has also been shown to be a putative substrate of BubR1 kinase activity, where CENP-E phosphorylation at S2639 by BubR1 promotes its ability to transition from lateral movement of the chromosome along microtubules to end-on association that tracks with the polymerizing plus-end of the microtubule [71]. However, it remains unexplored if T608 phosphorylation is involved in this process. In addition, studies have demonstrated experimentally that cells treated with the BubR1 kinase inhibitor Bubristatin, or mutations in the kinase domain predicted to render the kinase inactive, have misaligned chromosomes, suggesting that it has some importance in the activity occurring at kinetochores and/or in chromosome migration [43, 72, 73]. This is further cooperated by the observation that phosphorylation at T608 is sensitive to kinetochore-microtubule attachment state through its roles in targeting Mad2 to kinetochores, contributing to prolonged mitotic arrest, and promoting Aurora B-mediated phosphorylation of Ndc80 [59]. These results support that BubR1 may have a functional kinase domain that is important for its function in ensuring proper chromosome segregation. Given the proximity of T608 and T620, one possibility is that T608 is a target of Plk1 as it falls within a Plk1 phosphorylation consensus motif [37]. Likewise, it may be the target of another mitotic kinase and serve to promote Plk1 docking to BubR1.

Altogether, T608 phosphorylation seems to fill the role of polar chromosome rescue. It recruits SAC factors to lengthen mitotic arrest in their presence, whilst also promoting their ability to migrate. p-T608 thus provides a key reinforcement mechanism in maintaining genomic stability, without which small yet detrimental errors would be overlooked.

### Other identified phosphorylation sites

#### T792 and T1008

Threonine residues 792 and 1008 are located within the BubR1 kinase domain, and both have been identified as mitosis-specific phosphorylation sites. The use of various mutants of BubR1 at these sites has revealed that phosphorylation at T792 and T1008 appears to be intricately involved in chromosome alignment, but not SAC function [43, 73]. In addition, phosphorylation at these two sites may play a role in controlling BubR1 kinase activity. It has been considered that Plk1 is the kinase responsible for carrying out these phosphorylation events. The kinase domain of BubR1 contains four putative Plk1 phosphorylation consensus motifs. Phosphorylation-deficient mutants of BubR1 at amino acids in two of these four sites, T792 and T1008, were used in in vitro kinase assays with Plk1, which resulted in severely diminished phosphorylation compared to wild type BubR1, indicating that Plk1 has the ability to phosphorylate BubR1 at T792 and T1008 [43, 72, 73].

Interestingly, these PTMs may contribute to subsequent BubR1 phosphorylation events, particularly autophosphorylation [73]. Through kinase assays utilizing [γ-^32^P] ATP and various BubR1 mutants, it was found that phosphorylation-deficient amino acid substitutions at these sites result in decreased BubR1 autophosphorylation activity at T608 compared to wild type, while a phosphomimetic yielded markedly higher autophosphorylation levels. Together, these results suggest a potential role for Plk1 in regulating BubR1s alleged kinase activity. Phosphorylation at T792 and T1008 has been linked to chromosome alignment. Depletion of BubR1 yielded chromosome congression defects [73], and restoration of wild type or phosphomimetic substitutions of BubR1 rescued these observed defects, whereas a phosphorylation-deficient mutant was unable to improve chromosome alignment. Interestingly, a kinase-defective mutant still retaining phosphomimetic substitutions at T792 and T1008 showed decreased autophosphorylation activity and an inability to rescue chromosome alignment defects, further contributing to the notion of a functional kinase domain in BubR1. Given that localization of Plk1 contributes to chromosome congression [74], these studies suggest that phosphorylation of BubR1 at T792 and T1008 may have similar effects on BubR1 kinase activity that ultimately facilitate chromosome congression [73].

#### S1043

Less is known about phosphorylation of BubR1 at S1043, but certain aspects involving its timing and localization partners are beginning to be uncovered. In identifying non-Plk1 phosphorylation events on BubR1, phosphorylation at S1043 was shown to occur in conjunction with S670 [43]. Like S670, phosphorylation at S1043 is sensitive to kinetochore-microtubule attachments (as opposed to tension responsiveness) and occurs following nuclear envelope breakdown after BubR1 localizes to kinetochores [43]. Collective dephosphorylation of S670 and S1043 may take place after microtubule attachment to kinetochores just before anaphase onset [72].

Currently, there is no data that identifies BubR1 binding partners that require or relate to phosphorylated S1043. However, given the similarities of S1043 and S670 in phosphorylation patterns, it is possible that these residues have common interacting kinases/phosphatases. Cyclin B1-Cdk1 may phosphorylate S1043 to contribute to kinetochore-microtubule interactions. Further studies of S1043 are required to confirm its primary function and elucidate its relationship to S670, the kinase domain, and the various binding partners of BubR1 in the context of kinetochore-microtubule stability.

## Acetylation of BubR1

Acetylation is a well-characterized PTM that is abundant in the mammalian proteome, the vast majority taking place on lysine residues [75–77]. The enzymes responsible for the addition and removal of acetyl groups on *N*-ε-lysine residues are termed acetyltransferases and deacetylases, respectively [78]. This modification can also occur at the amino termini of polypeptides, and this process is thought to be irreversible. Acetylation neutralizes the positive charge of the amino group on lysine side chains, a feature that plays an important role in gene expression where it reversibly impacts the charge of histone tails protruding from the core nucleosome, influencing chromosome condensation and binding of transcription factors. Class I and II histone deacetylases (HDACs) regulate the expression and activity of numerous proteins involved in both cancer initiation and cancer progression. For instance, suppression of the cyclin-dependent kinase inhibitor p21 gene expression is associated with loss of acetylation of histones H3 and H4 in the p21 promoter region due to overexpression of HDACs in a variety of human tumors [79]. Sirtuin (SIRT)/class III deacetylases exert their function through deacetylation of various target proteins such as histones, α-tubulin [80], FOXO3a [81, 82], PPARγ [83] and p53 [84]. Interestingly, their activity is NAD^+^-dependent, suggesting that it is linked to the cellular metabolic state [85].

Although there are fewer reported BubR1 acetylation events compared to phosphorylation, they too play critical roles in the function of BubR1. Two significant sites of acetylation have been identified on BubR1, lysine 250 (K250) and lysine 668 (K668). Our current evidence of these sites suggests that they regulate BubR1 protein stability in opposite manners. Acetylation at K250 protects BubR1 from ubiquitination and premature degradation during mitosis [86], whereas acetylation at K668 promotes ubiquitin-mediated degradation of BubR1 during interphase [87].

### K250

The significance of acetylation on lysine 250 has been studied extensively and has been implicated both in the SAC and kinetochore-microtubule functions of BubR1 [88]. Cells deficient in K250 acetylation display severe alignment defects and aneuploidy, likely through compromising the timing of anaphase onset [86]. Acetylation of BubR1 at K250 is accomplished primarily by the acetyltransferase p300/CBP-associated factor (PCAF), whereas the reverse reaction is mediated by histone deacetylases HDAC2 and HDAC3. BRCA2 serves as a scaffold for BubR1 association with both PCAF and HDAC2/3 and appears to be required for these modifications to take place [89]. During prometaphase, PCAF acetylates K250, which protects BubR1 from ubiquitination and subsequent degradation by the 26S proteasome, thereby stabilizing BubR1s function until all chromosomes are attached and aligned correctly and the checkpoint is satisfied, at which point deacetylation of K250 is required for silencing the SAC. This promotes BubR1 ubiquitination and degradation, which diminishes the SAC response [86]. Further studies have indicated that the NAD^+^-dependent deacetylase SIRT2 can also target this site for deacetylation [90], though it remains unclear the temporal and spatial conditions that distinguish K250 regulation by each of the different deacetylases. K250 acetylation has also been implicated in BubR1s role in kinetochore-microtubule interactions and is proposed to enhance CENP-E and PP2A-B56 binding to BubR1 and recruitment to kinetochores [89].

Mechanistically, acetylation of K250 serves to protect BubR1 from becoming a substrate of APC/C^Cdc20^ [86]. Protected BubR1 thus interferes with the ability of APC/C^Cdc20^ to facilitate the ubiquitin-mediated degradation of various anaphase-inhibiting proteins, thereby keeping the cell arrested until all errors have been corrected and chromosome alignment has been successfully achieved. When K250 is deacetylated by HDAC2/3 (and potentially SIRT2) [89, 90], BubR1 relinquishes its role as an inhibitor and becomes a substrate of the APC/C^Cdc20^. Ubiquitination of BubR1 appears to cause it and the other components of the MCC to dissociate from each other and from the APC/C. With the various substrate recognition domains freed, the APC/C^Cdc20^ can go on to initiate anaphase. Mitosis is shortened in acetyl-deficient (K250R) expressing cells and prolonged in acetyl-mimetic (K250Q) expressing cells. Importantly, while expression of K250Q may promote chromosome congression and alignment, it may also prevent chromosomes from segregating, as cells with sustained K250 acetylation remain in metaphase for some time even after chromosome migration is complete and chromosomes are properly aligned [86, 88, 89]. As described in greater detail below, there may be additional PTMs of K250, such as SUMOylation, that may also be involved in regulating this process in addition to acetylation.

Less is known about the extent to which K250 acetylation is involved in BubR1s error correction function at kinetochores. Through the use of a heterozygous K250 acetylation-deficient mouse model (K243^R/+^ in mice), which shows high levels of aneuploidy and spontaneous tumor development [88], K250 acetylation was shown to have roles in both the SAC and in kinetochore-microtubule interactions. With respect to the latter, acetylated K250 enhances BubR1s involvement with both PP2A-B56 and CENP-E [88]. In the presence of proteasome inhibitors to maintain mitotic arrest, abolishing K250 acetylation reduced both PP2A-B56 and CENP-E binding to BubR1 and localization to kinetochores. This is consistent with the argument that CENP-E localization to kinetochores relies on its binding to BubR1 [88]. However, while cells with depleted CENP-E show a milder and more unique array of phenotypes, deficiency of K250 acetylation yields far more severe congression defects, which are rescued by expression of an acetyl-mimetic mutant. Thus, the degree of congression failure in acetylation-deficient mutants is likely not fully explained by the reduced binding and recruitment of CENP-E alone. Interestingly, K243^R/+^ mice displayed higher levels of Ndc80 phosphorylation than wild type controls, contradictory to p-T608, which relies on the BubR1-CENP-E relationship. This may be attributed to additional dysregulation of BubR1 function resulting from defective acetylation at K250.

### K668

K668 acetylation has been recognized as a significant BubR1 PTM during interphase. Opposite of K250, acetylation at K668 promotes BubR1 ubiquitination and degradation [87]. This PTM has been shown to be carried out by the acetyltransferase CREB-binding protein (CBP). The NAD^+^-dependent deacetylase SIRT2 reverses acetylation at K668, thereby protecting it from ubiquitination and degradation [87].

BubR1 has been implicated in the aging process, as evident by its levels declining naturally with age in a variety of tissues [13, 91–94]. Furthermore, a BubR1 hypomorphic mouse model, which expresses low levels of BubR1 from birth, exhibits extensive senescence and premature aging features [13]. Sirtuins, which are a family of NAD^+^-dependent deacetylases, are linked to protective mechanisms that delay the aging process and have been widely studied in protecting organisms from age-related pathogeneses [95]. SIRT2 is a sirtuin that has been shown to be associated with BubR1, and the availability of its cofactor NAD^+^ predicts the extent of its activity on BubR1. Like BubR1, NAD^+^ levels decline with age [96–98], suggesting that the drop in BubR1 levels over time may be driven by the loss of NAD^+^ and resulting decrease in SIRT2-mediated deacetylation of BubR1 at K668. Supporting this notion, restoring NAD^+^ in aged mice also restores BubR1 protein levels to those observed in young mice, which at the cellular level was largely dependent on SIRT2 [87].

Notably, the location of K668 within the KARD suggests that it may be a relevant BubR1 PTM during mitosis in addition to interphase. Its proximity to important KARD phosphorylation residues may implicate it in BubR1s kinetochore-microtubule function in error correction and chromosome migration, which requires further study.

## Ubiquitination of BubR1

Ubiquitination is a modification whereby the 76-amino-acid ubiquitin protein is covalently attached to the lysine residues of its target protein. The process of ubiquitination involves three catalytic steps utilizing a three-enzyme cascade consisting of an E1 ubiquitin-activating enzyme, an E2 ubiquitin-conjugating enzyme, and an E3 ubiquitin ligase enzyme that transfers the activated ubiquitin onto substrate proteins [99, 100]. Ubiquitination can serve as a signaling molecule when attached as a single moiety (monoubiquitination), but more often serves as a mechanism to target the protein to the 26S proteasome for degradation. The ubiquitin–proteasome pathway is a regulated cellular process that signals the degradation of specific proteins for the purpose of maintaining homeostasis. This is achieved through recognition domains on target proteins, which consist of specific sequence motifs for binding to ubiquitin–proteasome pathway machinery.

While there is clear evidence of ubiquitination occurring on BubR1 and that it influences many BubR1 functions and roles in disease, there is little understanding of the specifics such as the enzymes involved or the target lysines on BubR1. During mitosis, Cdc20 and BubR1 ubiquitination appears to promote their dissociation from other MCC components and the APC/C, ultimately freeing the APC/C and its substrate recognition subunit Cdc20 to degrade mitotic proteins and initiate anaphase [101]. While it remains unclear whether BubR1s ubiquitin-mediated degradation is required for anaphase and mitotic exit to take place, there is evidence that BubR1 requires ubiquitination to dissociate from the APC/C even in cells absent of the proteasome. This suggests the possibility that BubR1 degradation is not required for anaphase to take place, but its ubiquitination-mediated disassociation from the MCC is [102]. It inevitably becomes targeted to the 26S proteasome, however, once it has dissociated from the MCC. This is reflected in the cell cycle-dependent variation in BubR1 levels (i.e., high in G2 and M, low in G1 and S). BubR1 is also targeted for ubiquitination during interphase, which may be important in regulating BubR1 abundance as organisms age. However, further studies are necessary to fully understand the regulation of BubR1 ubiquitination that would dictate these processes of aneuploidy and aging.

Target protein specificity is determined primarily by the numerous E3 ligases. In the context of BubR1, mitotic-dependent ubiquitination is likely carried out by the APC/C, which recognizes its substrates by their KEN boxes, D boxes, TPR motifs, and ABBA motifs of which BubR1 has many (Fig. 1) [30, 103]. The E3 ligase responsible for K668 acetylation-dependent BubR1 degradation during interphase, however, remains unknown.

Though BubR1 possesses ample recognition sequences for ubiquitination machinery and is a confirmed target of the ubiquitin–proteasome pathway, exactly which lysines on BubR1 serve as ubiquitin acceptors remain unknown. The mechanisms that determine lysine site specificity (if any) for ubiquitination on a designated target protein remain poorly understood in general, and methods of identification of specific lysine residue targets for ubiquitination are only now becoming better characterized. Therefore, further work is necessary to fully illuminate the ubiquitination and degradation processes controlling BubR1 stability, as well as if there are deubiquitinating enzymes (DUBs) that target ubiquitinated BubR1 to control its functions during mitosis and/or aging.

## SUMOylation of BubR1

Small Ubiquitin-like Modifier (SUMO) proteins are similar to ubiquitin, though they are not specifically used as tags for degradation. SUMOylation occurs on lysine residues [104] and is a rapidly reversible process with numerous cellular functions, including transcriptional regulation. SUMO proteins are added to their targets in a manner similar to ubiquitination, involving E1, E2, and E3 enzymes [104]. In humans, there are four known SUMO isoforms, simply named SUMO-1, SUMO-2, SUMO-3, and SUMO-4 [104, 105].

Although SUMOylation of BubR1 has been described, its role in regulating BubR1 function has yet to be fully elucidated. K250 is currently the only known lysine residue on BubR1 to be targeted for SUMOylation [106]. Of the four vertebrate SUMO isoforms, BubR1 is likely modified primarily by SUMO-2, but may also be modified by SUMO-1 [106] and SUMO-3 [107]. The E3 enzyme responsible for BubR1 SUMOylation remains unidentified, although the RanBP2 E3 SUMO ligase is known to be associated with SUMOylation of other mitotic proteins and has been shown to be involved in chromosome segregation [106]. BubR1 SUMOylation has been identified as important in the context of kinetochore-microtubule activity, and SUMO-deficient mutants exhibit prolonged mitotic delay and errors in chromosome segregation.

As previously discussed, the residue modified by SUMOylation on BubR1 also serves as an acceptor site for acetylation during mitosis. Acetylation of BubR1 at K250 is required during early phases of mitosis and present throughout chromosome congression while the SAC is active, then reversed to allow SUMOylation at K250 to take place, which may be important in mitotic exit. Consistent with this, Acetyl-K250 and SUMO-K250 levels were inversely correlated in metaphase cells with aligned chromosomes [108]. SUMOylation of BubR1 appears to be required for BubR1 to dissociate from kinetochores post-congression, which was necessary for chromatids to properly separate from each other upon anaphase onset. SUMO-deficient BubR1 remained enriched abnormally at the kinetochores of aligned chromosomes, whereas wild type BubR1 had since dispersed. Surprisingly, SUMO-deficiency was associated with an increased number of “ring” chromosomes and dicentric chromosomes still linked by their telomeres [108]. Compared to wild type, the separation of chromatids at the centromere occurred prematurely, but progression through the remainder of mitosis was delayed due to persistent kinetochore localized BubR1 hindering their inability to separate completely and remaining linked at the telomeres. These observations suggest that SUMO-K250 is required for BubR1 to dissociate from kinetochores, which has a role in full separation of sister chromatids and timely segregation. These results also call into question a role for BubR1 in telomere cohesion. Although it is generally known and accepted that the cohesin complex is responsible for sister telomere linkage and is removed in early mitosis due to the action of separase, the presence of ring chromosomes in BubR1 SUMO-deficient cells introduces additional questions regarding BubR1s involvement in this process. Telomeres of sister chromatids have been shown to be linked to each other by tethers independent of the cohesin complex [109, 110]. Therefore, BubR1 may be associated with these tethers, as it has also been shown to associate with DNA tethers present in acentric chromosomes [111]. However, the structural composition of these telomeric tethers remains unknown [109]. In addition, BubR1 interacts with Sgo1, a protein involved in maintaining centromere cohesion. These studies have demonstrated that BubR1 appears to interact with unphosphorylated, interphase Sgo1 and that SUMOylation at K250 may mediate this interaction [108].

Importantly, linking the consequences of deficient and mimetic amino acid substitutions of K250 to an individual PTM is difficult, as this site is subject to multiple modifications. Thus far, we know K250 undergoes both acetylation and SUMOylation and may be subject to ubiquitination. It is important to note the use of K250R and K250Q substitutions in many of these studies of both acetylated and SUMOylated K250. K250R is both an acetyl-deficient and SUMO-deficient mutant. Thus, the phenotypes resulting from this substitution may not reflect the loss of a single PTM but rather the combined loss of multiple PTMs. In addition, the acetyl-mimetic K250Q may mimic acetylation but is consequently also SUMO-deficient. These factors make the study of K250 and other such sites a challenge and reinforce that identifying the enzymes responsible for the addition and removal of SUMO at K250 will be important in addressing the roles of these PTMs in regulating BubR1 function.

## Other PTMs on BubR1

In addition to phosphorylation, acetylation, ubiquitination, and SUMOylation, there are well over 200 other potential modifications [39, 40], some of which no doubt occur on BubR1 but have yet to be identified. It is likely that BubR1 is targeted by other important PTMs such as glycosylation, succinylation and hydroxylation. Another common PTM, methylation, takes place primarily on arginine and lysine residues and is a reversible process carried out by methyltransferases and demethylases. Methylation frequently competes with lysine acetylation, especially in the context of gene expression, where it has the opposite effect by promoting chromatin compaction and inhibiting transcription [112]. According to the Phosphosite database (www.phosphosite.org), di-methylation of BubR1 was found in mass spectrometry analysis on residue Arginine-919. However, a role for this modification has yet to be discerned, and further studies are necessary to deduce the purpose of methylation, as well as other common PTMs, on BubR1.

## Observations in PTM cooperativity

**Fig. 4:**
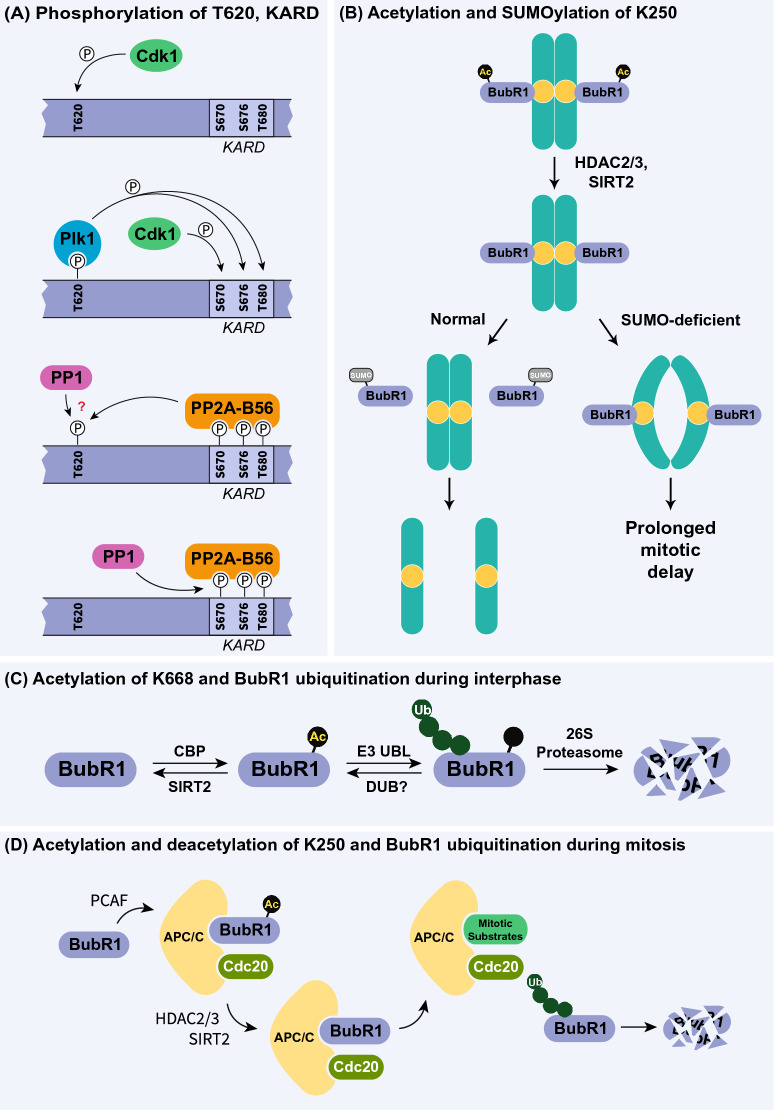
PTM crosstalk on BubR1. **a** Sequential phosphorylation of BubR1 at T620, followed by KARD phosphorylation and PP2A-B56 binding during mitosis. **b** SUMOylation of BubR1 regulating chromatid separation during anaphase. **c** Lysine-668 Acetylation-dependent ubiquitination and degradation of BubR1 during interphase. **d** Acetylation and deacetylation of K250 during the SAC

Crosstalk between different PTMs is a crucial mechanism in increasing the diversity of functional consequences associated with PTMs on a given protein. Transcription, cell cycle regulation, protein stability and DNA damage responses are a few processes that rely heavily on cooperativity and/or competition between PTMs. BubR1 is no exception to this idea (Fig. 4). For example, simultaneous mutation of S543, S574, S670, S720, and S1043 either to all phospho-deficient or all phosphomimetic substitutions revealed extreme deficits in chromosome attachment, congression, and alignment, with phenotypes almost identical to total BubR1 depletion [42]. In addition, a phospho-deficient/mimetic of these sites excluding S670 (which had already previously been identified as a major contributor to these processes) yielded major defects as well, demonstrating the significant contribution of these other four sites. This may point to the requirement for rapid reversibility of PTMs at these particular sites throughout the process of chromosome migration, maintaining the “push–pull” effect necessary for these processes. However, the role of each of these sites individually, as well as their role in regulating BubR1 function together, requires further evaluation.

Many of the PTMs on BubR1 described above cooperatively regulate BubR1 function (Fig. 4). One important area of PTM cooperativity is observed in the relationship between T620 phosphorylation and the events that follow it during mitotic onset. Serving as a key initiation site, p-T620 creates a binding surface for Plk1 to dock and subsequently phosphorylate BubR1, at sites such as the KARD, promoting recruitment of the PP2A-B56 phosphatase. An area of cooperativity between different post-translational modifications that reside on the same residue is observed in the relationship between acetylation and SUMOylation of K250. The data thus far suggest a sequential regulation of BubR1 through different modifications to K250 in which proper mitotic timing requires K250 deacetylation at kinetochores to allow for K250 SUMOylation to take place. Relatedly, this points to a possible molecular switch between SAC activation and terminal SAC silencing that is reliant on the occurrence of this sequential modification of K250. However, as described above, results utilizing deficient and mimetic amino acid substitutions of K250 to study either acetylation or SUMOylation should be evaluated in the context of how the mutation impacts both modifications and the resulting alteration to BubR1 function. Furthermore, the literature regarding acetylation of K250 is discussed largely in the context of BubR1 SAC function, which is not exclusively carried out on kinetochores. Meanwhile, the literature on SUMOylated BubR1 is largely discussed in the context of BubR1s presence at kinetochores. To fully understand the complex regulation of BubR1 by multiple PTMs at K250 as well as sequential PTM regulation at regions such as the KARD will require further investigation, especially with respect to their spatial and temporal regulation and consequences to BubR1 functionality. Furthermore, these modifications will also need to be assessed in the context of their importance to genomic integrity, cancer, aging, and MVA.

## Conclusions

As of now, much of the mechanistic basis of BubR1-mediated pathophysiology remains ambiguous. For instance, it is still poorly understood how MVA-related BubR1 mutations shape BubR1 function to promote aneuploidy. Similarly, it remains unclear how somatic mutations and PTMs modulate BubR1 to control genomic integrity as well as cellular senescence and the aging process. This is further complicated by our incomplete knowledge of the functionality of many other PTMs, including those that have yet to be discovered on BubR1, as well as the full complexity of the BubR1 domain structure having yet to be deciphered. Much progress has been made in determining how the BubR1 domain structure coordinates its interactive and PTM-receptive functionality (Table 2), but certain important aspects such as the purpose of the kinase domain and the exact roles of the numerous degron motifs and the IC20BD (internal Cdc20 binding domain) remain to be further defined in depth [32]. Importantly, the 3D crystal structure of BubR1 has not been fully elucidated, likely due to its size and the potential unstructured nature of many of its linker regions. This is a significant obstacle, as protein conformation is critical in our understanding of how PTMs across the protein may cooperate to control BubR1 function.

**Table 2:** Characterized PTMs occurring on BubR1

Modification	Residue	Added by	Reversed by	Mechanism	Functional consequences	Mutational consequences	References
Phosphorylation	T608	BubR1 (auto) Plk1?	PP1?	Unknown—acts on Aurora B kinase	Polar chromosome migration SAC reinforcement	Deficient: reduced Aurora B-mediated KMN phosphorylation, attachment errors	[59]
	T620	Cdk1	PP2A-B56, PP1-Knl1	Docking site for Plk1	KC-MT stability	Deficient: defective KC-MT attachments, prolonged mitotic arrest	[28, 46]
	S670	Cdk1 Mps1? Other?	PP1-Knl1	Recruit PP2A-B56	KC-MT stability (attachment sensitive)	Deficient: loss of tension downstream? High incidence of lagging chromosomes Chromosome alignment defects	[42, 43, 113]
	S676	Plk1	PP1-Knl1	Recruit PP2A-B56	KC-MT stability (tension sensitive)	Deficient: chromosome alignment defects	[28, 37, 45, 113]
	T680	Plk1	PP1-Knl1	Recruit PP2A-B56	KC-MT stability (tension sensitive)	Deficient: chromosome alignment defects	[37, 45, 113]
	T792	Plk1	Unknown	Unknown	BubR1 kinase activity KC-MT stability	Deficient: decreased BubR1 autophosphorylation Mimetic: Increased BubR1 autophosphorylation	[43, 73]
	T1008	Plk1	Unknown	Unknown	BubR1 kinase activity KC-MT stability	Deficient: decreased BubR1 phosphorylation Mimetic: increased BubR1 phosphorylation	[43, 73]
	S1043	Mps1 Cdk1?	Unknown	Unknown	Unknown (attachment sensitive, PTM patterns resemble S670)	–	[43]
Acetylation	K250	PCAF	HDAC2/3 SIRT2	Protect BubR1 from premature degradation during mitosis by blocking APC/C	MCC stability, anaphase inhibition Chromosome migration	K250R (deficient): accelerated mitosis, missegregation of chromosomes K250Q (mimetic): delayed chromosome segregation following alignment	[86, 88–90]
	K668	CBP	SIRT2	Signal ubiquitination machinery	BubR1 ubiquitination, degradation during interphase	Deficient: Increased BubR1 abundance	[87]
Ubiquitination	Unknown	Cdc20-APC/C	Unknown	Target BubR1 to proteasome	Dissociation of MCC BubR1 degradation	–	[101, 102]
SUMOylation	K250	Unknown RanBP2 SUMO E3 ligase?	Unknown	Dissociation of BubR1 from kinetochore; association with CENP-E; facilitate BubR1-Sgo1 interaction; BubR1 degradation?	BubR1 degradation? SAC silencing (checkpoint inactivation) Timely and complete chromosome segregation	Deficient: BubR1 retained on kinetochores, anaphase delay and premature chromatid separation	[106, 108]

Further exploration of BubR1 with respect to PTMs, domain structure, and binding partners will shed more light on BubR1 function within the cell, and how dysfunction of these processes leads genomic instability, aging, and age-related diseases such as cancer.

## Data Availability

Not applicable.
